# Associations between twelve composite inflammatory indices and sarcopenia in a health examination population: a cross-sectional study

**DOI:** 10.3389/fpubh.2026.1838379

**Published:** 2026-06-12

**Authors:** Li Zhang, Na Shen, Qian Xiao, Kang Luo, Zhiwen Yan, Cheng Luo

**Affiliations:** 1 Department of Geriatrics, Laboratory of Research and Translation for Geriatric Diseases, The First Affiliated Hospital of Chongqing Medical University, Chongqing, China; 2 Department of Oncology, Yunyang County People's Hospital, Chongqing, China; 3 Department of Critical Care Medicine, The First Affiliated Hospital of Chongqing Medical University, Chongqing, China; 4 Department of Neurology, The First Affiliated Hospital of Chongqing Medical University, Chongqing, China

**Keywords:** adults, early screening, health examination population, inflammatory indices, sarcopenia

## Abstract

**Background:**

Sarcopenia is strongly linked to chronic inflammation, yet most studies have focused on individual inflammatory markers. Moreover, associations between inflammatory status and sarcopenia have not been systematically compared in health examination populations. This study explored the relationships between composite inflammatory indices and sarcopenia in individuals undergoing routine health examinations.

**Methods:**

This retrospective cross-sectional study included 2617 participants from a health examination cohort. Composite inflammatory indices were derived from peripheral blood cell counts and standard biochemical parameters. Independent associations between each index and sarcopenia were examined using multivariable logistic regression. Subgroup analyses by sex and age were conducted, restricted cubic spline models assessed potential non-linear relationships, and receiver operating characteristic curves were used to compare discriminative performance.

**Results:**

Among 2,617 participants, sarcopenia prevalence was 5.5%. After adjustment for confounders, higher inflammatory status was associated with increased sarcopenia risk. The C-reactive protein-to-lymphocyte ratio (CLR) showed the strongest association (Q4 vs. Q1: OR = 4.31, 95% CI 2.35–7.90). Higher levels of the C-reactive protein-to-albumin ratio (CAR), systemic immune-inflammation index (SII), and platelet-to-lymphocyte ratio (PLR) were also significantly associated with higher sarcopenia risk. Associations were consistent across sex and age subgroups (all interaction *p* > 0.05). Discriminative performance was modest; the hemoglobin–albumin–lymphocyte–platelet score (HALP) (AUC = 0.637), PLR (AUC = 0.623), and CLR (AUC = 0.618) performed relatively better, while combining multiple indices did not significantly improve prediction.

**Conclusion:**

Several composite inflammatory indices derived from routine laboratory data are associated with sarcopenia risk in health examination populations and may provide additional information for assessing inflammation-related sarcopenia risk.

## 1 Introduction

Sarcopenia is a progressive skeletal muscle disease characterized by a generalized loss of skeletal muscle mass, declining muscle strength, and deterioration in physical function (1, 2). As the global population ages rapidly, sarcopenia has emerged as a significant risk factor for frailty, falls, disability, and mortality among older adults, imposing considerable medical and societal burdens on families and healthcare systems (3–5). Of particular concern, adverse lifestyle patterns—including prolonged sedentary time, insufficient physical activity, and nutritional imbalance—often coexist with metabolic abnormalities, potentially shifting muscle decline to earlier stages of life. Consequently, the risk of sarcopenia has been rising among younger and middle-aged adults (6–8). Therefore, advancing the window for identification and intervention through early surveillance and prevention is crucial for healthy aging and for maintaining skeletal muscle health across the life course.

Chronic low-grade inflammation is a key biological pathway in the development and progression of sarcopenia (9, 10). Pro-inflammatory cytokines may impair skeletal muscle homeostasis by suppressing anabolic signaling involved in protein synthesis while activating catabolic pathways, thereby accelerating muscle wasting (11–13). Prior research indicates a strong correlation between elevated traditional inflammatory biomarkers, including C-reactive protein (CRP) and interleukin-6 (IL-6), and rapid declines in muscle mass and physical performance in older adults (11, 14, 15). Recently, composite inflammatory indices derived from peripheral blood cell counts and routine biochemical parameters, including the C-reactive protein-to-albumin ratio (CAR), platelet-to-lymphocyte ratio (PLR), and systemic immune-inflammation index (SII), have garnered increasing attention. By integrating information on inflammatory burden, immune status, and nutritional condition, these indices may provide more stable and clinically accessible inflammatory phenotypes than single markers alone (16, 17). Although they have been widely applied in oncology and cardiovascular research (18, 19), large-scale and systematic evidence remains limited regarding their associations with sarcopenia risk in health examination populations—particularly in full-spectrum adult cohorts that include young and middle-aged individuals.

This study systematically assessed the relationships between twelve composite inflammatory indices and sarcopenia risk in adults aged 30 years and above using a large health examination dataset. We further compared the discriminative performance of these indices to identify markers potentially associated with sarcopenia status.

## 2 Materials and methods

### 2.1 Study design and participants

The research utilized a cross-sectional design. Participants were adults undergoing routine health exams at Chongqing Medical University’s First Affiliated Hospital Health Examination Center from November 2017 to November 2019. Participants were eligible if they were 30 years or older and capable of completing the questionnaire.

Participants were excluded if they had: (1) severe limb disability, hemiplegia, or psychiatric disorders affecting ambulation or handgrip strength testing; (2) severe organic diseases like malignant tumors, end-stage renal disease, or liver cirrhosis; (3) medications affecting muscle metabolism or inflammation, such as glucocorticoids or immunosuppressants; (4) acute infection (WBC > 10 × 10^9^/L) or COPD exacerbation; or (5) missing key blood test or physical examination data required for sarcopenia assessment and primary analyses. During the study period, 3,353 examinees who met the age criterion were initially screened. A total of 736 participants were excluded, including 669 due to missing blood test data and 67 due to missing physical examination data. A total of 2,617 participants were included in the final analysis. Baseline characteristics between included and excluded participants were additionally compared (Supplementary Table S1), and no significant difference in sarcopenia prevalence was observed between the two groups.

The study adhered to the Declaration of Helsinki and received approval from the Medical Ethics Committee of the First Affiliated Hospital of Chongqing Medical University (approval No. 2022-K460). This cross-sectional study adhered to the STROBE guidelines for reporting observational research (20). Participants voluntarily enrolled and gave written informed consent.

### 2.2 Measurement of inflammatory biomarkers and calculation of inflammatory indices

Venous blood samples were collected from all participants after a minimum 8-h overnight fast and analyzed at the Clinical Laboratory of the First Affiliated Hospital of Chongqing Medical University. Complete blood counts and selected biochemical parameters were measured using standardized laboratory procedures. This study examined twelve composite inflammatory indices derived from peripheral blood parameters. The laboratory variables comprised neutrophil (N), lymphocyte (L), and monocyte (M) counts, platelet count (P), hemoglobin (Hb) and serum albumin (Alb) levels, and high-sensitivity C-reactive protein (hs-CRP).

Twelve composite indices representing systemic inflammation and nutritional status were systematically derived from primary hematological and biochemical measurements (21–23). The indices examined included the C-reactive protein–albumin–lymphocyte index (CALLY), C-reactive protein-to-albumin ratio (CAR), C-reactive protein-to-lymphocyte ratio (CLR), hemoglobin–albumin–lymphocyte–platelet score (HALP), monocyte-to-lymphocyte ratio (MLR), neutrophil-to-lymphocyte ratio (NLR), neutrophil-to-platelet ratio (NPR), platelet-to-albumin ratio (PAR), platelet-to-lymphocyte ratio (PLR), pan-immune-inflammation value (PIV), systemic immune-inflammation index (SII), and systemic inflammation response index (SIRI). The specific formulas were defined as follows:


CALLY=Alb×L/(hs−CRP×10)

CAR=hs−CRP/Alb

CLR=hs−CRP/L

HALP=Hb×Alb×L/P

MLR=M/L

NLR=N/L

NPR=N/P

PAR=P/Alb

PLR=P/L

PIV=N×M×P/L

SII=P×N/L

SIRI=N×M/L

### 2.3 Definition of sarcopenia

Sarcopenia was identified following the 2019 Asian Working Group for Sarcopenia (AWGS) guidelines, which evaluate skeletal muscle mass, muscle strength, and physical performance (24).

Appendicular skeletal muscle mass (ASM) was measured using bioelectrical impedance analysis (BIA; TANITA MC-780, Japan). The appendicular skeletal muscle mass index (ASMI) was determined by dividing the appendicular skeletal muscle mass (ASM) by the square of the height (kg/m^2^) to adjust for body size variations. Low muscle mass was defined as an ASMI < 7.0 kg/m^2^ for men and < 5.7 kg/m^2^ for women.

Muscle strength was evaluated through handgrip strength (HGS) using a Jamar hydraulic hand-held dynamometer. Participants stood upright with a neutral forearm and wrist position during testing and were instructed to grip the dynamometer with maximum force. Each hand was tested twice, and the mean of the highest values from both tests was used for further analysis. Low muscle strength is characterized by handgrip strength (HGS) below 28 kg for men and below 18 kg for women.

Physical performance was evaluated using 4-meter gait speed (GS). Participants walked at their normal pace from start to finish, with time measured to the nearest 0.01 s using a stopwatch. A gait speed below 1.0 m/s indicated impaired physical function. The 4-meter gait speed test has also been widely used in epidemiological and clinical studies and has demonstrated good performance in reflecting physical function among Chinese adults and older populations (25).

Sarcopenia, as per AWGS 2019 criteria, is identified by low skeletal muscle mass along with either diminished muscle strength or decreased physical performance.

### 2.4 Covariates

Covariates were selected based on prior literature, potential physiological mechanisms, and clinical relevance to minimize the potential influence of confounding factors on the study findings (26, 27). Information on covariates was obtained through structured questionnaires and standardized physical measurements, covering demographic characteristics, socioeconomic status, living conditions, lifestyle behaviors, and medical history. All questionnaires and physical assessments were conducted by well-trained healthcare professionals following unified protocols. To minimize potential human error and ensure data quality, a double independent data entry system with cross-validation was implemented. The questionnaire gathered data on: (1) Demographics: age, sex, ethnicity (Han/non-Han), and education level (< high school, high school, > high school); (2) Socioeconomic and living conditions: live alone (yes/no), employment status (yes/no), and monthly expenditure (<1,000; 1,000–3,000; 3,000–6,000; >6,000); (3) Lifestyle factors: dietary habit (light/spicy), smoking (yes/no), alcohol use (yes/no), and physical activity. Physical activity levels were evaluated using the International Physical Activity Questionnaire (IPAQ) and classified as low, moderate, or high based on standard scoring procedures (28). Additionally, medical history was reviewed, focusing on hypertension and diabetes. Trained personnel measured body weight and height, and calculated body mass index (BMI) as weight divided by height squared (kg/m^2^). All covariates had <5% missing values and were imputed using the K nearest neighbors (KNN) method.

### 2.5 Statistical analysis

Baseline characteristics were summarized after stratification by sarcopenia status (sarcopenia vs. non-sarcopenia). Normally distributed continuous variables were presented as mean ± standard deviation (mean ± SD). Non-normally distributed continuous variables were presented as medians and compared between groups using the Mann–Whitney U test. Categorical variables were represented as counts and percentages [*n* (%)] and analyzed using the chi-square test. To systematically assess the relationship between twelve composite inflammatory indices and sarcopenia risk, each index was divided into quartiles, with Q1 (≤25th percentile) as the reference group, followed by Q2 (25th–50th percentile), Q3 (50th–75th percentile), and Q4 (>75th percentile). Multivariable logistic regression models were used to estimate odds ratios (ORs) and 95% confidence intervals (CIs). Three models with varying levels of adjustment were developed: Model 1 was unadjusted; Model 2 included adjustments for age, sex, ethnicity, educational level, living alone status, employment status, and monthly expenditure; Model 3 incorporated additional adjustments for dietary habit, body mass index (BMI), smoking, alcohol consumption, physical activity, diabetes, and hypertension, alongside the covariates in Model 2. Subgroup and interaction analyses were performed according to age (<65 vs. ≥65 years) and sex. Restricted cubic spline (RCS) models with four knots at the 5th, 35th, 65th, and 95th percentiles were used to explore potential dose–response relationships and evaluate non-linear associations. The discriminative ability of the twelve inflammatory indices for identifying sarcopenia was assessed using receiver operating characteristic (ROC) curves and the area under the curve (AUC). Based on AUC values, the three indices with the best diagnostic performance were selected to construct a combined marker, and the discriminative performance of single indices versus the combined marker was further compared. Differences in AUCs across ROC curves were assessed using the DeLong test. A two-sided *p* value less than 0.05 was deemed statistically significant. Statistical analyses were performed using R software (v4.2.2).

## 3 Results

### 3.1 Comparison of baseline characteristics

Table 1 summarizes the baseline characteristics of the study participants. A total of 2,617 individuals were included and were classified according to the AWGS 2019 criteria into a non-sarcopenia group (*n* = 2,473) and a sarcopenia group (*n* = 144). Participants with sarcopenia showed significant differences in sex, BMI, physical activity, employment status, smoking, and alcohol consumption compared to those without the condition (all *p* < 0.05). The sarcopenia group exhibited a higher percentage of men (65.28% vs. 47.88%, *p* < 0.001), as well as increased rates of smoking (22.22% vs. 15.12%, *p* = 0.022) and alcohol consumption (33.33% vs. 23.78%, *p* = 0.009) compared to the non-sarcopenia group. Participants with sarcopenia had a significantly lower mean BMI (20.7 ± 2.5 kg/m^2^) compared to those without (24.2 ± 2.9 kg/m^2^, *p* < 0.001). The distribution of physical activity levels varied significantly between groups (*p* = 0.009), with the sarcopenia group exhibiting a higher percentage of individuals engaging in high physical activity (29.17% compared to 21.55%) and a lower percentage in low physical activity (8.33% compared to 16.70%). With respect to employment, a greater percentage of participants with sarcopenia were employed (57.64% vs. 48.04%, *p* = 0.025). The prevalence of hypertension showed no significant difference between groups (*p* = 0.080). No cases of diabetes were observed in the sarcopenia group, whereas the prevalence of diabetes in the non-sarcopenia group was 2.59%, and this difference reached statistical significance (*p* = 0.048). The groups showed no significant differences in age, ethnicity, educational attainment, income level, dietary habit, or living alone status (all *p* > 0.05).

**Table 1:** Baseline characteristics of study subjects classified by sarcopenia status.

Sarcopenia					
Characteristic	Overall *N* = 2,617	No *N* = 2,473	Yes *N* = 144	Statistic^1^	*p*-value
Age, mean ± SD	54.5 ± 9.2	54.5 ± 9.2	54.6 ± 9.2	−0.04	0.970^2^
Sex, *n* (%)				16.49	< 0.001^3^
Male	1,278 (48.83%)	1,184 (47.88%)	94 (65.28%)		
Female	1,339 (51.17%)	1,289 (52.12%)	50 (34.72%)		
Ethnicity, n (%)					0.580^4^
No-han	63 (2.41%)	61 (2.47%)	2 (1.39%)		
Han	2,554 (97.59%)	2,412 (97.53%)	142 (98.61%)		
Education, *n* (%)				0.65	0.724^3^
< High school	1,078 (41.19%)	1,021 (41.29%)	57 (39.58%)		
High school	798 (30.49%)	756 (30.57%)	42 (29.17%)		
> High school	741 (28.31%)	696 (28.14%)	45 (31.25%)		
BMI, mean ± SD	24.0 ± 3.0	24.2 ± 2.9	20.7 ± 2.5	15.75	< 0.001^2^
Live alone, *n* (%)				0.25	0.617^3^
No	2,465 (94.19%)	2,328 (94.14%)	137 (95.14%)		
Yes	152 (5.81%)	145 (5.86%)	7 (4.86%)		
Employment status, *n* (%)				5.02	0.025^3^
No	1,346 (51.43%)	1,285 (51.96%)	61 (42.36%)		
Yes	1,271 (48.57%)	1,188 (48.04%)	83 (57.64%)		
Monthly expenses, *n* (%)					0.092^4^
< 1,000	640 (24.46%)	615 (24.87%)	25 (17.36%)		
1,000–3,000	1,223 (46.73%)	1,154 (46.66%)	69 (47.92%)		
3,000–6,000	692 (26.44%)	644 (26.04%)	48 (33.33%)		
˃ 6,000	62 (2.37%)	60 (2.43%)	2 (1.39%)		
Dietary habit, *n* (%)				0.00	0.955^3^
Light	287 (10.97%)	271 (10.96%)	16 (11.11%)		
Spicy	2,330 (89.03%)	2,202 (89.04%)	128 (88.89%)		
Physical activity, *n* (%)				9.47	0.009^3^
Low	425 (16.24%)	413 (16.70%)	12 (8.33%)		
Med	1,617 (61.79%)	1,527 (61.75%)	90 (62.50%)		
High	575 (21.97%)	533 (21.55%)	42 (29.17%)		
Smoke, *n* (%)				5.23	0.022^3^
No	2,211 (84.49%)	2,099 (84.88%)	112 (77.78%)		
Yes	406 (15.51%)	374 (15.12%)	32 (22.22%)		
Drink, *n* (%)				6.76	0.009^3^
No	1,981 (75.70%)	1,885 (76.22%)	96 (66.67%)		
Yes	636 (24.30%)	588 (23.78%)	48 (33.33%)		
Hypertension, *n* (%)				3.07	0.080^3^
No	2,384 (91.10%)	2,247 (90.86%)	137 (95.14%)		
Yes	233 (8.90%)	226 (9.14%)	7 (4.86%)		
Diabetes, *n* (%)					0.048^4^
No	2,553 (97.55%)	2,409 (97.41%)	144 (100.00%)		
Yes	64 (2.45%)	64 (2.59%)	0 (0.00%)		
hsCRP, mean ± SD	1.37 ± 2.09	1.35 ± 1.96	1.81 ± 3.62	*t* = −1.55	0.1231^2^

^1^Welch two sample *t*-test; Pearson’s chi-squared test; fisher’s exact test.

^2^welch two sample *t*-test.

^3^Pearson’s chi-squared test.

^4^Fisher’s exact test.

SD, Standard deviation; BMI, Body mass index.

### 3.2 Associations between inflammatory indices and sarcopenia

We developed three logistic regression models to analyze the associations between the twelve inflammatory indices and sarcopenia risk (Table 2). The unadjusted model showed significant associations between various composite inflammatory indices and sarcopenia. Sarcopenia risk escalated with higher quartiles of CAR, CLR, MLR, NLR, PLR, SII, and SIRI, with all trends showing statistical significance (*p* < 0.05). In contrast, higher levels of CALLY (P for trend < 0.001) and HALP (P for trend < 0.001) were associated with a significantly lower risk of sarcopenia. In the unadjusted analyses, no significant associations were found for NPR, PAR, or PIV, as all *p*-values for trend exceeded 0.05. After adjustment for demographic and socioeconomic variables in Model 2, these overall patterns remained largely consistent. CAR, CLR, CALLY, HALP, MLR, NLR, PLR, and SII maintained significant associations with sarcopenia (all P for trend < 0.05), while the association for SIRI weakened (P for trend = 0.249). In the fully adjusted Model 3, increased levels of CAR, CLR, MLR, NLR, PLR, and SII were independently linked to greater odds of sarcopenia, whereas elevated CALLY and HALP were inversely related to sarcopenia risk (all P for trend < 0.05). In Model 3, participants in the highest CAR quartile (Q4) exhibited significantly greater odds of sarcopenia compared to those in the lowest quartile (Q1) (OR = 2.82, 95% CI 1.62–4.89; *p* < 0.001; P for trend < 0.001). The most significant positive correlation was found for CLR, with an odds ratio of 4.31 (95% CI: 2.35–7.90) when comparing the highest quartile (Q4) to the lowest quartile (Q1), and both the *p*-value and the P for trend were less than 0.001. Higher NLR and PLR in Q4 were linked to an increased risk of sarcopenia, with NLR showing an OR of 1.69 (95% CI 1.01–2.85; *p* = 0.047; P for trend = 0.029) and PLR an OR of 1.81 (95% CI 1.09–3.01; *p* = 0.022; P for trend < 0.001). For SII, the OR for Q4 versus Q1 was 2.15 (95% CI: 1.28–3.62; *p* = 0.004; P for trend = 0.004). Notably, MLR also demonstrated a robust positive association. The adjusted odds ratios (ORs) for MLR, compared to Q1, were 2.11 (95% CI: 1.13–3.94; *p* = 0.020) for Q2, 1.98 (95% CI: 1.06–3.71; *p* = 0.033) for Q3, and 3.21 (95% CI: 1.76–5.86; *p* < 0.001) for Q4, with a significant trend (*p* < 0.001). CALLY and HALP both demonstrated significant inverse relationships with sarcopenia, with CALLY in the fourth quartile and HALP each associated with substantially lower odds (OR = 0.22, 95% CI 0.12–0.42 for CALLY and 0.13–0.37 for HALP; *p* < 0.001; P for trend < 0.001). In Model 3, NPR, PAR, and PIV showed no significant association with sarcopenia (all P for trend > 0.05).

**Table 2:** Association between the four quartiles of 12 composite inflammatory biomarkers and the risk of sarcopenia.

Sarcopenia	Cases/Controls	Model 1 OR (95% CI); *p*-value	Model 2 OR (95% CI); *p*-value	Model 3 OR (95% CI); *p*-value
CALLY quartile				
Q1 (Ref.)	54/668	1.00	1.00	1.00
Q2	39/668	0.70 (0.46, 1.08); 0.108	0.73 (0.47, 1.12); 0.151	0.73 (0.47, 1.12); 0.150
Q3	43/668	0.78 (0.52, 1.19); 0.247	0.78 (0.51, 1.19); 0.249	0.77 (0.50, 1.18); 0.228
Q4	13/669	0.23 (0.12, 0.42); < 0.001	0.23 (0.12, 0.43); < 0.001	0.22 (0.12, 0.42); < 0.001
*P* for trend		< 0.001	< 0.001	< 0.001
CAR quartile				
Q1 (Ref.)	19/668	1.00	1.00	1.00
Q2	41/668	2.23 (1.28, 3.89); 0.005	2.19 (1.25, 3.82); 0.006	2.32 (1.33, 4.07); 0.003
Q3	40/668	2.18 (1.25, 3.80); 0.006	2.19 (1.25, 3.83); 0.006	2.31 (1.31, 4.06); 0.004
Q4	49/669	2.70 (1.57, 4.64); < 0.001	2.61 (1.51, 4.51); < 0.001	2.82 (1.62, 4.89); < 0.001
*P* for trend		< 0.001	< 0.001	< 0.001
CLR quartile				
Q1 (Ref.)	14/668	1.00	1.00	1.00
Q2	39/668	2.90 (1.56, 5.39); < 0.001	2.84 (1.53, 5.30); 0.001	2.90 (1.55, 5.42); < 0.001
Q3	40/668	2.98 (1.60, 5.52); < 0.001	3.02 (1.62, 5.62); < 0.001	3.09 (1.65, 5.76); < 0.001
Q4	56/669	4.27 (2.35, 7.74); < 0.001	4.19 (2.29, 7.65); < 0.001	4.31 (2.35, 7.90); < 0.001
*P* for trend		< 0.001	< 0.001	< 0.001
HALP quartile				
Q1 (Ref.)	64/668	1.00	1.00	1.00
Q2	40/668	0.60 (0.40, 0.91); 0.015	0.50 (0.33, 0.77); 0.001	0.53 (0.35, 0.81); 0.004
Q3	24/668	0.35 (0.22, 0.57); < 0.001	0.25 (0.15, 0.42); < 0.001	0.27 (0.16, 0.44); < 0.001
Q4	21/669	0.31 (0.18, 0.51); < 0.001	0.20 (0.12, 0.34); < 0.001	0.22 (0.13, 0.37); < 0.001
*P* for trend		< 0.001	< 0.001	< 0.001
MLR quartile				
Q1 (Ref.)	17/668	1.00	1.00	1.00
Q2	36/668	2.18 (1.21, 3.93); 0.009	2.01 (1.12, 3.63); 0.020	2.11 (1.13, 3.94); 0.020
Q3	38/668	2.31 (1.29, 4.13); 0.005	2.03 (1.13, 3.67); 0.018	1.98 (1.06, 3.71); 0.033
Q4	58/669	3.64 (2.09, 06.31); < 0.001	3.01 (1.71, 5.31); < 0.001	3.21 (1.76, 5.86); < 0.001
*P* for trend		< 0.001	< 0.001	< 0.001
NLR quartile				
Q1 (Ref.)	29/668	1.00	1.00	1.00
Q2	33/668	1.15 (0.69, 1.91); 0.603	1.07 (0.64, 1.79); 0.797	1.07 (0.62, 1.88); 0.801
Q3	35/668	1.22 (0.74, 2.02); 0.443	1.12 (0.67, 1.85); 0.674	1.29 (0.74, 2.24); 0.371
Q4	52/669	1.86 (1.16, 2.96); 0.009	1.66 (1.03, 2.67); 0.036	1.69 (1.01, 2.85); 0.047
*P* for trend		0.008	0.030	0.029
NPR quartile				
Q1 (Ref.)	52/669	1.00	1.00	1.00
Q2	47/668	0.80 (0.51, 1.24); 0.314	0.78 (0.50, 1.22); 0.286	0.76 (0.46, 1.25); 0.277
Q3	38/668	0.66 (0.42, 1.06); 0.084	0.65 (0.41, 1.04); 0.072	0.67 (0.40, 1.12); 0.129
Q4	32/668	0.66 (0.42, 1.05); 0.082	0.66 (0.41, 1.05); 0.079	0.69 (0.41, 1.17); 0.170
*P* for trend		0.054	0.107	0.136
PAR quartile				
Q1 (Ref.)	35/665	1.00	1.00	1.00
Q2	30/670	0.84 (0.51, 1.39); 0.505	0.87 (0.52, 1.43); 0.578	0.88 (0.53, 1.47); 0.629
Q3	39/667	1.12 (0.70, 1.79); 0.642	1.19 (0.74, 1.92); 0.465	1.19 (0.74, 1.92); 0.473
Q4	45/671	1.29 (0.82, 2.04); 0.267	1.51 (0.95, 2.40); 0.080	1.54 (0.97, 2.46); 0.069
*P* for trend		0.151	0.116	0.088
PIV quartile				
Q1 (Ref.)	36/668	1.00	1.00	1.00
Q2	27/668	0.74 (0.44, 1.23); 0.247	0.66 (0.40, 1.11); 0.121	0.67 (0.40, 1.12); 0.126
Q3	43/668	1.21 (0.77, 1.91); 0.417	1.05 (0.66, 1.67); 0.830	1.07 (0.67, 1.71); 0.772
Q4	43/669	1.21 (0.76, 1.90); 0.421	1.02 (0.64, 1.62); 0.942	1.08 (0.68, 1.74); 0.741
*P* for trend		0.166	0.497	0.168
PLR quartile				
Q1 (Ref.)	29/668	1.00	1.00	1.00
Q2	16/668	0.54 (0.29, 1.01); 0.052	0.56 (0.30, 1.04); 0.068	0.54 (0.28, 1.04); 0.067
Q3	45/668	1.59 (0.99, 2.57); 0.058	1.73 (1.06, 2.80); 0.027	1.46 (0.86, 2.46); 0.162
Q4	59/669	2.13 (1.35, 3.37); 0.001	2.39 (1.50, 3.81); < 0.001	1.81 (1.09, 3.01); 0.022
*P* for trend		< 0.001	< 0.001	< 0.001
SII quartile				
Q1 (Ref.)	30/668	1.00	1.00	1.00
Q2	27/668	0.90 (0.53, 1.52); 0.685	0.87 (0.51, 1.48); 0.604	1.16 (0.65, 2.08); 0.613
Q3	41/668	1.39 (0.86, 2.26); 0.181	1.34 (0.83, 2.19); 0.235	1.83 (1.07, 3.13); 0.028
Q4	51/669	1.76 (1.10, 2.79); 0.018	1.73 (1.08, 2.76); 0.022	2.15 (1.28, 3.62); 0.004
*P* for trend		0.004	0.005	0.004
SIRI quartile				
Q1 (Ref.)	31/668	1.00	1.00	1.00
Q2	32/668	1.03 (0.62, 1.71); 0.897	0.92 (0.55, 1.53); 0.75	1.16 (0.67, 2.02); 0.595
Q3	37/668	1.20 (0.74, 1.97); 0.456	1.01 (0.61, 1.67); 0.962	1.34 (0.78, 2.32); 0.29
Q4	49/669	1.62 (1.02, 2.58); 0.04	1.28 (0.79, 2.07); 0.322	1.83 (1.07, 3.13); 0.026
*P* for trend		0.027	0.249	0.156

Model 1 was unadjusted.

Model 2 was adjusted for age, sex, ethnicity, educational level, living alone, employment status, and monthly expenses.

Model 3 was further adjusted for dietary habit, BMI, smoking status, alcohol consumption, physical activity, diabetes, and hypertension.

### 3.3 Subgroup analyses

Subgroup analyses stratified by sex and age (<65 years and ≥65 years) were conducted to evaluate the robustness of the associations between the twelve composite inflammatory indices and sarcopenia risk, aiming to minimize potential heterogeneity due to demographic characteristics (Supplementary Table S2). The findings demonstrated consistent associations between inflammatory indices and sarcopenia across various subgroups, indicating stable predictive value regardless of sex or age. Formal interaction tests showed no significant effects between sex or age and inflammatory indices (all *p* > 0.05), suggesting that sex and age did not significantly alter the relationship between composite inflammatory indices and sarcopenia risk.

### 3.4 Non-linear associations between inflammatory indices and sarcopenia

**Figure 1:**
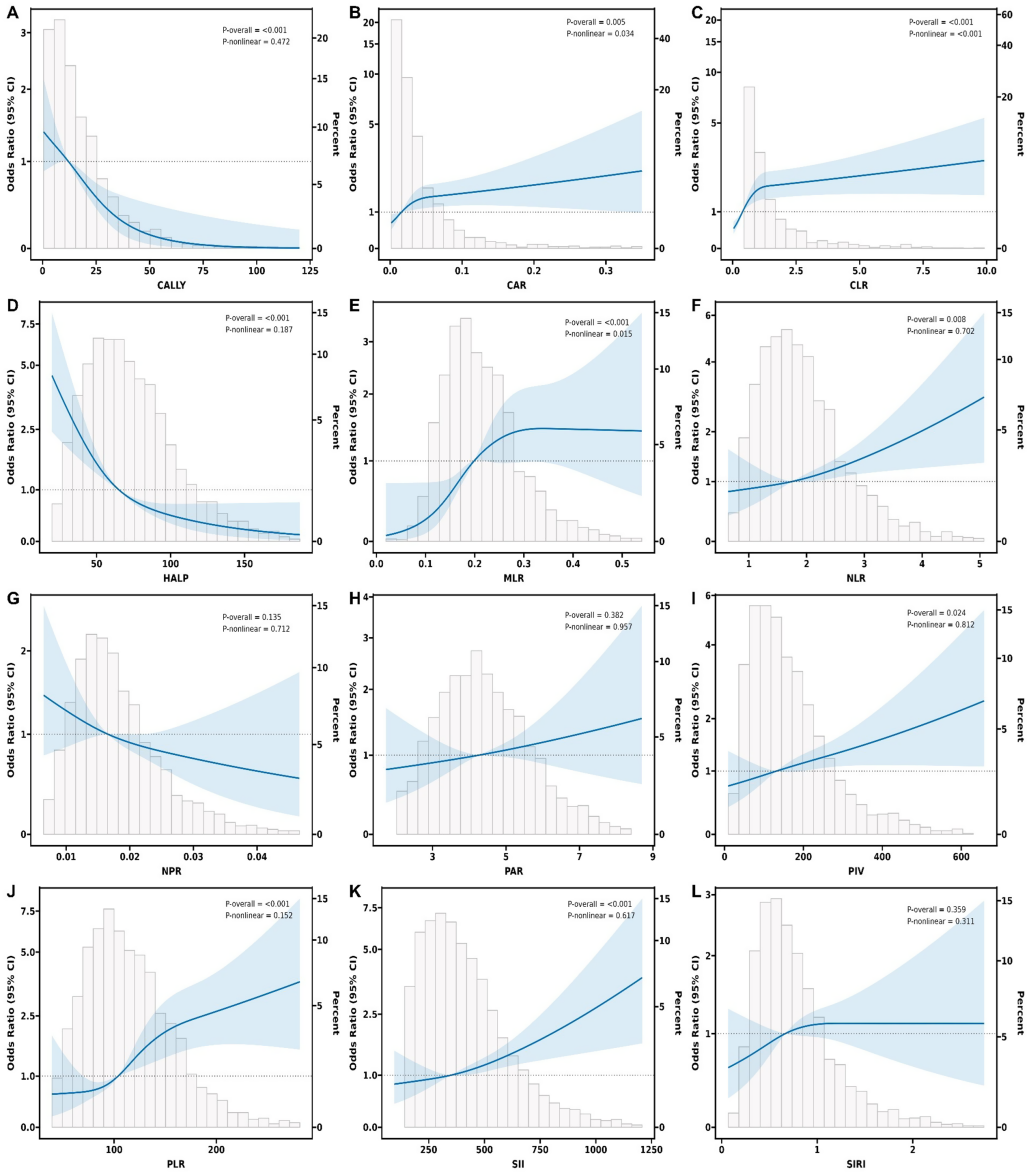
Dose–response relationships between twelve composite inflammatory indices and the risk of sarcopenia. Note: Restricted cubic spline (RCS) models were used to depict adjusted odds ratios (ORs; solid lines) and their 95% confidence intervals (shaded areas). The gray histograms represent the frequency distribution of each inflammatory index among participants. *P* for overall indicates the significance of the overall association, and *P* for non-linearity indicates the significance of the non-linear trend. Panels correspond to the following indices: **(A)** CALLY, **(B)** CAR, **(C)** CLR, **(D)** HALP, **(E)** MLR, **(F)** NLR, **(G)** NPR, **(H)** PAR, **(I)** PIV, **(J)** PLR, **(K)** SII, and **(L)** SIRI.

Figure 1 illustrates the dose–response relationships between twelve composite inflammatory indices and sarcopenia risk using restricted cubic spline (RCS) models. CALLY and HALP demonstrated significant inverse relationships with sarcopenia risk (P for overall < 0.001), with no significant non-linearity detected (P for non-linearity = 0.472 and 0.187, respectively), suggesting primarily linear associations. In contrast, several indices reflecting inflammatory burden—including CAR, CLR, MLR, NLR, PIV, PLR, and SII—were positively associated with sarcopenia risk (all P for overall < 0.05). Significant non-linear dose–response relationships were identified for CAR (*p* = 0.034), CLR (*p* < 0.001), and MLR (*p* = 0.015), indicating potential non-linear associations. The non-linearity tests for NLR (*p* = 0.702), PIV (*p* = 0.812), PLR (*p* = 0.152), and SII (*p* = 0.617) were not statistically significant, indicating that their risk relationships likely follow linear trends. No significant associations with sarcopenia risk were found for NPR (*p* = 0.135), PAR (*p* = 0.382), or SIRI (*p* = 0.359), and their non-linearity tests were also non-significant (*p* = 0.712, 0.957, and 0.311, respectively).

### 3.5 Discriminative performance of inflammatory indices for identifying sarcopenia

**Figure 2:**
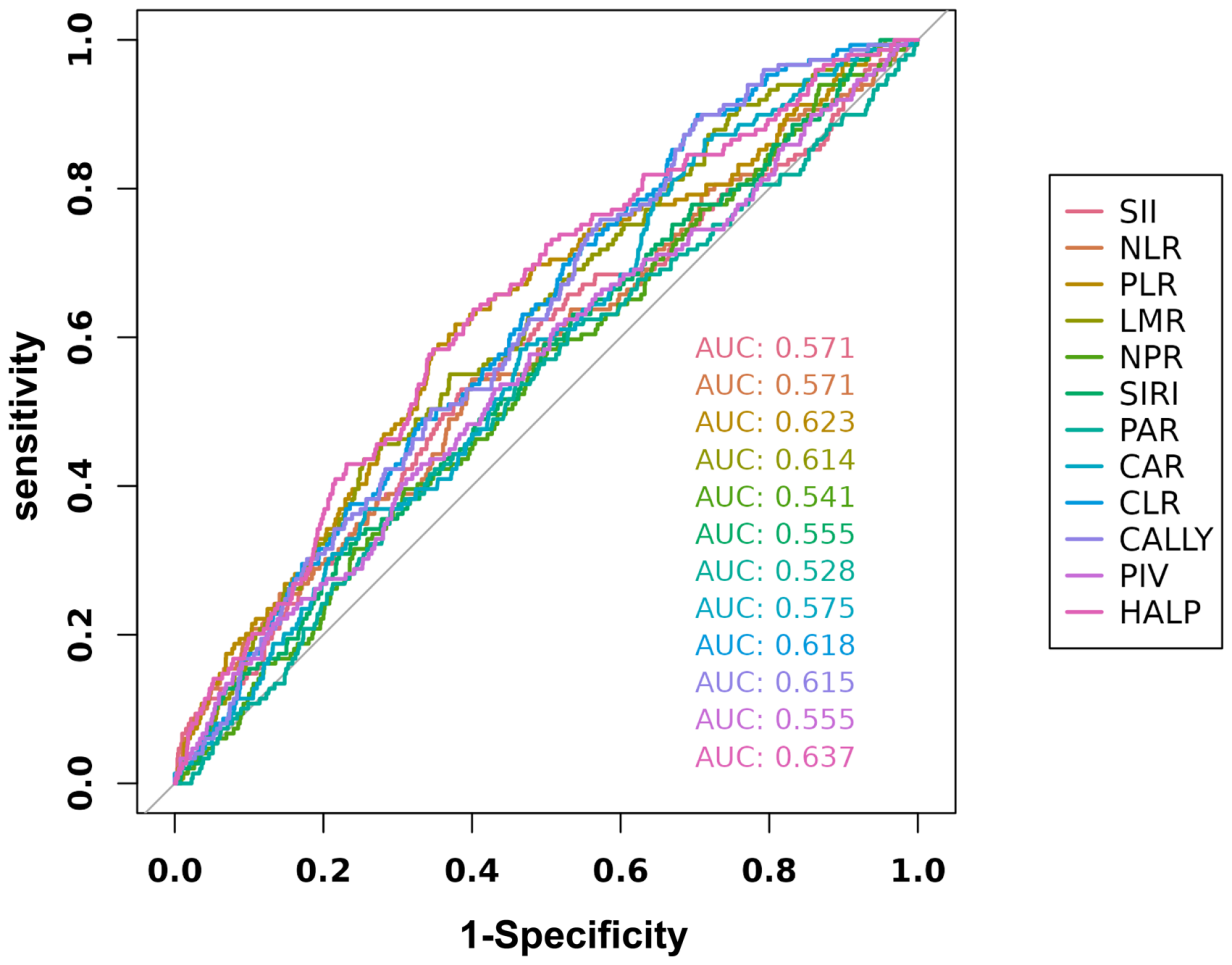
ROC curves for identifying sarcopenia risk using 12 composite inflammatory markers. The figure presents the area under the curve (AUC) value for each inflammatory marker, with the colored labels corresponding to the ROC curves in the same top-to-bottom order. The diagonal line represents random prediction (AUC = 0.5).

**Figure 3:**
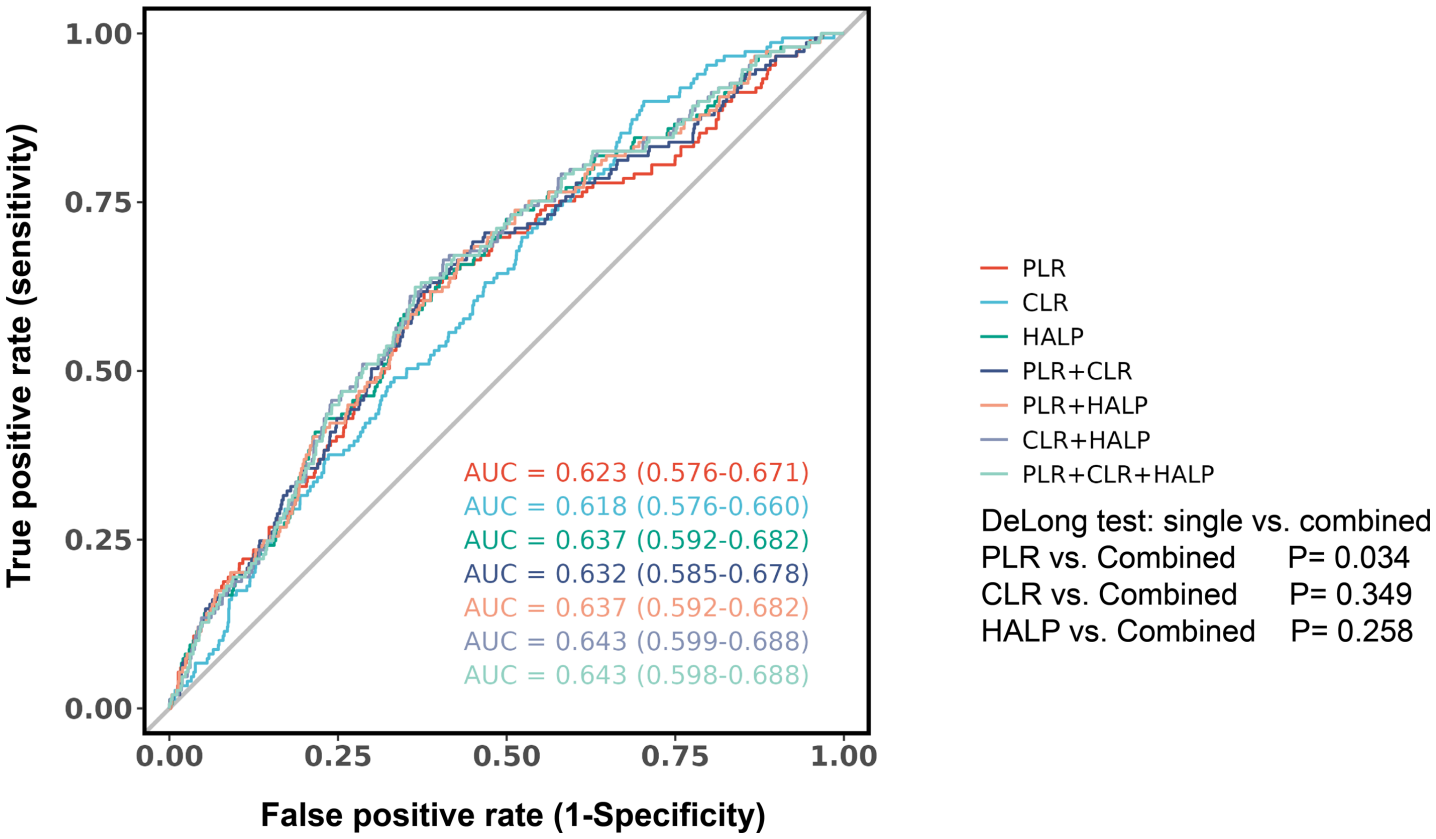
ROC curves of the top three inflammatory markers (PLR, CLR, HALP) with AUC and their combined model for identifying sarcopenia risk. The *x*-axis represents 1 − specificity (false positive rate), and the *y*-axis represents sensitivity (true positive rate). The figure presents the area under the curve (AUC) and corresponding 95% confidence interval (CI) for each marker and combined model, with the colored labels corresponding to the ROC curves in the same top-to-bottom order. The diagonal line represents the random prediction result (AUC = 0.5).

Receiver operating characteristic (ROC) curves were generated for all twelve composite inflammatory indices to assess their ability to discriminate sarcopenia, and the areas under the curve (AUCs) were calculated (Figure 2). The indices demonstrated low to moderate discriminative capacity, with AUCs between 0.528 and 0.637. Among them, HALP demonstrated the highest discrimination (AUC = 0.637), followed by PLR (AUC = 0.623) and CLR (AUC = 0.618). The three indices with the highest AUCs were then selected to construct combined prediction models (Figure 3). In the combined analyses, the AUCs were 0.632 (95% CI: 0.585–0.678) for PLR + CLR, 0.637 (95% CI: 0.592–0.682) for PLR + HALP, and 0.643 (95% CI: 0.599–0.688) for CLR + HALP. The integrated model of PLR, CLR, and HALP indices achieved an AUC of 0.643 (95% CI 0.598–0.688). DeLong tests showed that the three-index model did not significantly differ from HALP (*p* = 0.258) or CLR (*p* = 0.349) in terms of AUC, and was only marginally superior to PLR (*p* = 0.034). Collectively, these results suggest that HALP, PLR, and CLR offer relatively better discriminative performance for sarcopenia in a health examination population, whereas combining multiple indices provides limited incremental benefit over the best single marker, HALP.

## 4 Discussion

This study systematically evaluates the associations between twelve composite inflammatory indices and sarcopenia risk using a large health examination cohort. Our study reveals a significant association between various indices of systemic chronic inflammatory burden, such as CAR, CLR, MLR, NLR, PLR, and SII, and the heightened likelihood of sarcopenia. In contrast, composite indices capturing both nutritional and inflammatory status (e.g., CALLY and HALP) showed significant inverse associations with sarcopenia risk. Subgroup analyses and interaction tests further supported the robustness of these associations across sex and age strata. RCS analyses suggested that some inflammatory indices may exhibit non-linear relationships with sarcopenia risk. In ROC analyses, HALP, PLR, and CLR demonstrated relatively better discriminative performance, whereas combining multiple indices did not meaningfully enhance predictive ability. These findings provide epidemiological evidence supporting associations between routinely derived composite inflammatory indices and sarcopenia in health examination populations. The observed associations may contribute to a better understanding of the relationship between systemic inflammation and sarcopenia.

A substantial body of evidence has established chronic low-grade inflammation as an important pathological mechanism underlying muscle mass loss and functional decline (24, 29). Epidemiological studies across diverse populations have also reported close links between systemic inflammation-related composite indices and sarcopenia risk. For example, one cross-sectional study observed significant positive associations of SII, SIRI, and MLR with sarcopenia, with SII showing relatively strong discriminative value (30). A community-based study of Thai adults aged 60 and above also identified a correlation between elevated SIRI and MLR levels and an increased risk of sarcopenia (31). Existing evidence predominantly addresses older adults (≥60 years) or hospitalized patients, with limited systematic data from health examination populations. By comprehensively assessing these associations in a health examination cohort aged ≥30 years, our study helps address this knowledge gap. Moreover, whereas many prior studies examined only one or a small number of indices, we compared twelve composite inflammatory indices in parallel, thereby providing broader epidemiological support for understanding the inflammation–sarcopenia relationship.

Chronic low-grade inflammation is recognized as a fundamental pathological basis for sarcopenia, engaging intricate, multi-level mechanisms across various physiological systems. Pro-inflammatory cytokines, such as TNF-*α*, IL-6, and IL-1, released during inflammation, can disrupt muscle protein homeostasis by promoting protein degradation via ubiquitin–proteasome pathways and inhibiting anabolic signaling like mTOR, leading to myofiber atrophy and muscle mass reduction (9). In parallel, inflammatory mediators can activate canonical pathways such as NF-κB, further amplifying catabolic signaling and perpetuating a sustained muscle-wasting state (32). Second, chronic inflammation often interacts with oxidative stress, leading to mitochondrial dysfunction and impaired energy metabolism; these changes increase susceptibility to myocyte apoptosis and injury and may accelerate declines in muscle strength and physical function (33, 34). In addition, in the context of aging, cellular senescence and immunosenescence-related inflammation can maintain persistently elevated pro-inflammatory cytokine levels, creating a vicious cycle between inflammation and muscle deterioration (32, 35). Notably, in metabolically adverse conditions such as sarcopenic obesity, adipose tissue can release pro-inflammatory mediators and exacerbate insulin resistance, further increasing systemic inflammatory burden and accelerating muscle wasting (36). Taken together, inflammation may contribute to sarcopenia not only through direct effects on protein catabolism and energy dysregulation, but also by interacting with aging and metabolism-related pathways. These mechanisms offer biologically plausible explanations for our observation that multiple composite inflammatory indices were strongly associated with sarcopenia risk, and they further suggest that interventions targeting inflammatory burden—such as exercise, dietary optimization, and therapies aimed at inflammatory pathways—may have value in sarcopenia prevention and management.

Our RCS analyses showed that most inflammatory indices (e.g., NLR, PIV, PLR, and SII) exhibited approximately linear positive associations with sarcopenia risk. Although a few indices (CAR, CLR, and MLR) demonstrated statistically significant non-linear patterns, the overall trend still indicated increasing risk with higher index levels. Consistently, subgroup analyses and interaction testing suggested that these associations were stable across sex and age categories, supporting the robustness of the relationship between inflammatory burden and sarcopenia. ROC analyses further indicated that PLR, CLR, and HALP had some potential for discriminating sarcopenia, although the AUC values (0.618–0.637) suggest that overall predictive performance remains limited. While DeLong tests indicated statistically significant differences between some combined and single-index models, the magnitude of improvement was small, implying that inflammation-related indices alone may be insufficient for substantially improving prediction accuracy. Our systematic evaluation therefore provides an important basis for future efforts to develop more efficient prediction models by integrating multi-dimensional measures (e.g., body composition, nutritional status, and lifestyle factors) alongside inflammatory indices.

This research possesses multiple strengths. First, the large health examination sample covered the full adult age spectrum—from young and middle-aged adults to older individuals—enhancing representativeness. Second, by systematically comparing twelve inflammatory indices, we reduced the narrow perspective inherent in single-index investigations. Third, the analytical framework was rigorous, incorporating multivariable adjustment, RCS-based non-linearity assessment, subgroup analyses, ROC evaluation, and DeLong testing, which collectively strengthen the reliability of our conclusions. Nevertheless, several limitations should be acknowledged. Due to the cross-sectional design, establishing causal relationships and temporal sequences between inflammatory indices and sarcopenia is not possible; confirmation requires prospective longitudinal studies. Second, the recruitment of participants from a single center may introduce regional or selection bias, potentially limiting the generalizability of the findings. Differences in population characteristics compared with previous community-based or older adult(s) cohorts may also partly explain inconsistencies in sarcopenia prevalence patterns across studies. Third, the limitations of routine health examinations prevented us from conducting parallel comparisons with circulating cytokines like IL-6 and TNF-*α*. Ultimately, despite adjusting for various covariates, the possibility of residual confounding from unmeasured factors remains. Future multicenter prospective cohort studies are warranted to further clarify the longitudinal associations between these composite inflammatory indices and sarcopenia, particularly in young and middle-aged populations.

## 5 Conclusion

In summary, this study provides robust evidence that multiple composite inflammatory indices—particularly HALP, CLR, and PLR—are significantly associated with sarcopenia risk in a health examination population. These associations remained stable across sex and across adult age groups. Composite inflammatory indices derived from routinely available peripheral blood parameters may provide accessible and clinically relevant information for evaluating inflammation-related sarcopenia risk and could contribute to a better understanding of the relationship between systemic inflammation and sarcopenia.

## Data Availability

The raw data supporting the conclusions of this article will be made available by the authors, without undue reservation.
