# Supplementary material for: Associations between twelve composite inflammatory indices and sarcopenia in a health examination population: a cross-sectional study
Source: Front Public Health. 2026 Jun 12;14:1838379. doi: 10.3389/fpubh.2026.1838379 (PMC13303356; doi:10.3389/fpubh.2026.1838379)
Supplement: Supplementary file 1 [file Table_1.DOCX]

**Table S1: Baseline characteristics of included and excluded participants.**

| **Characteristic** | **Group** | | **p-value** |
| --- | --- | --- | --- |
|  | **Included participants  N = 2,617** | **Excluded participants  N =736** |  |
| **Age, Mean ± SD** | 54.5 ± 9.2 | 55.39 ± 10.48 | 0.427^1^ |
| **Sex, n (%)** |  |  | <0.001^2^ |
| Male | 1,278 (48.83%) | 433 (58.82%) |  |
| Female | 1,339 (51.17%) | 303 (41.18%) |  |
| **Ethnicity, n (%)** |  |  | 0.592^2^ |
| No-han | 63 (2.41%) | 20 (2.71%) |  |
| Han | 2,554 (97.59%) | 716 (97.29%) |  |
| **Education, n (%)** |  |  | 0.006^2^ |
| < High School | 1,078 (41.19%) | 271 (36.80%) |  |
| High School | 798 (30.49%) | 212 (28.81%) |  |
| > High School | 741 (28.31%) | 253 (34.39%) |  |
| **Live alone, n (%)** |  |  | 0.978^2^ |
| No | 2,465 (94.19%) | 43 (5.88%) |  |
| Yes | 152 (5.81%) | 693 (94.12%) |  |
| **Employment status, n (%)** |  |  | 0.741^2^ |
| No | 1,346 (51.43%) | 355 (48.27%) |  |
| Yes | 1,271 (48.57%) | 381 (51.73%) |  |
| **Monthly expenses, n (%)** |  |  | 0.002^2^ |
| ˂ 1000 | 640 (24.46%) | 148 (20.06%) |  |
| 1000-3000 | 1,223 (46.73%) | 326 (44.34%) |  |
| 3000-6000 | 692 (26.44%) | 245 (33.33%) |  |
| ˃ 6000 | 62 (2.37%) | 17 (2.26%) |  |
| **Spicy food, n (%)** |  |  | 0.769^2^ |
| No | 287 (10.97%) | 80 (10.86%) |  |
| Yes | 2,330 (89.03%) | 656 (89.14%) |  |
| **Smoke, n (%)** |  |  | 0.977^2^ |
| No | 2,211 (84.49%) | 623 (84.62%) |  |
| Yes | 406 (15.51%) | 113 (15.38%) |  |
| **Drink, n (%)** |  |  | 0.611^2^ |
| No | 1,981 (75.70%) | 552 (74.96%) |  |
| Yes | 636 (24.30%) | 184 (25.04%) |  |
| **BMI, Mean ± SD** | 24.0 ± 3.0 | 24.06 ± 3.02 | 0.241^1^ |
| **Physical activity, n (%)** |  |  | 0.069^2^ |
| Low | 425 (16.24%) | 94 (12.82%) |  |
| Med | 1,617 (61.79%) | 462 (62.75%) |  |
| High | 575 (21.97%) | 180 (24.43%) |  |
| **Hypertension, n (%)** |  |  | 0.038^2^ |
| No | 2,384 (91.10%) | 648 (88.08%) |  |
| Yes | 233 (8.90%) | 88 (11.92%) |  |
| **Diabetes, n (%)** |  |  | 0.354^2^ |
| No | 2,553 (97.55%) | 712 (96.68%) |  |
| Yes | 64 (2.45%) | 24 (3.32%) |  |
| **Sarcopenia, n (%)** |  |  | 0.283^2^ |
| No | 2,524 (94.43%) | 703 (95.48%) |  |
| Yes | 149 (5.57%) | 33 (4.52%) |  |
| ^1^Welch Two Sample t-test | | | |
| ^2^Pearson's Chi-squared test | | | |

**Table S2: Subgroup analysis of the association between 12 inflammatory markers and sarcopenia (by age and sex)**

| **Subgroup** | **N** | **OR (95% CI)** | **P value** | **P for interaction** |
| --- | --- | --- | --- | --- |
| **CALLY** | 2673 | 0.957 (0.941-0.974) | <0.001 |  |
| Age |  |  |  | 0.603 |
| < 65 | 1841 | 0.960 (0.941-0.980) | <0.001 |  |
| ≥ 65 | 832 | 0.952 (0.919-0.986) | 0.006 |  |
| Sex |  |  |  | 0.995 |
| Male | 1318 | 0.960 (0.938-0.981) | <0.001 |  |
| Female | 1355 | 0.956 (0.929-0.984) | 0.002 |  |
| **CAR** | 2673 | 40.328 (3.445-472.048) | 0.003 |  |
| Age |  |  |  | 0.416 |
| < 65 | 1841 | 9.609 (0.092-1001.843) | 0.340 |  |
| ≥ 65 | 832 | 68.673 (3.208-1470.094) | 0.007 |  |
| Sex |  |  |  | 0.300 |
| Male | 1318 | 16.179 (0.893-292.956) | 0.060 |  |
| Female | 1355 | 303.517 (2.231-41299.233) | 0.023 |  |
| **CLR** | 2673 | 1.141 (1.036-1.256) | 0.007 |  |
| Age |  |  |  | 0.716 |
| < 65 | 1841 | 1.115 (0.943-1.319) | 0.204 |  |
| ≥ 65 | 832 | 1.142 (1.011-1.289) | 0.032 |  |
| Sex |  |  |  | 0.169 |
| Male | 1318 | 1.102 (1.005-1.207) | 0.038 |  |
| Female | 1355 | 1.287 (1.066-1.555) | 0.009 |  |
| **HALP** | 2673 | 0.984 (0.977-0.993) | <0.001 |  |
| Age |  |  |  | 0.452 |
| < 65 | 1841 | 0.985 (0.976-0.995) | 0.003 |  |
| ≥ 65 | 832 | 0.983 (0.968-0.999) | 0.036 |  |
| Sex |  |  |  | 0.093 |
| Male | 1318 | 0.976 (0.965-0.986) | <0.001 |  |
| Female | 1355 | 0.986 (0.971-1.000) | 0.056 |  |
| **MLR** | 2673 | 35.205 (5.852-211.782) | <0.001 |  |
| Age |  |  |  | 0.421 |
| < 65 | 1841 | 12.971 (1.079-155.952) | 0.043 |  |
| ≥ 65 | 832 | 57.933 (3.768-890.624) | 0.004 |  |
| Sex |  |  |  | 0.345 |
| Male | 1318 | 10.685 (0.966-118.187) | 0.053 |  |
| Female | 1355 | 117.999 (5.338-2608.431) | 0.003 |  |
| **NLR** | 2673 | 1.385 (1.152-1.665) | 0.001 |  |
| Age |  |  |  | 0.056 |
| < 65 | 1841 | 1.192 (0.937-1.516) | 0.153 |  |
| ≥ 65 | 832 | 1.753 (1.277-2.406) | 0.001 |  |
| Sex |  |  |  | 0.576 |
| Male | 1318 | 1.273 (1.010-1.604) | 0.041 |  |
| Female | 1355 | 1.534 (1.099-2.142) | 0.012 |  |
| **NPR** | 2673 | 0.000 (0.000-19.031) | 0.082 |  |
| Age |  |  |  | 0.412 |
| < 65 | 1841 | 0.000 (0.000-0.115) | 0.037 |  |
| ≥ 65 | 832 | 0.000 (0.000-36339043261.610) | 0.475 |  |
| Sex |  |  |  | 0.309 |
| Male | 1318 | 0.000 (0.000-0.001) | 0.016 |  |
| Female | 1355 | 0.001 (0.000-72778301919271536.000) | 0.753 |  |
| **PAR** | 2673 | 1.067 (0.937-1.214) | 0.330 |  |
| Age |  |  |  | 0.486 |
| < 65 | 1841 | 1.048 (0.891-1.233) | 0.570 |  |
| ≥ 65 | 832 | 1.160 (0.936-1.438) | 0.174 |  |
| Sex |  |  |  | 0.732 |
| Male | 1318 | 1.113 (0.944-1.313) | 0.203 |  |
| Female | 1355 | 1.035 (0.832-1.287) | 0.760 |  |
| **PIV** | 2673 | 1.002 (1.001-1.003) | 0.005 |  |
| Age |  |  |  | 0.280 |
| < 65 | 1841 | 1.001 (0.999-1.003) | 0.217 |  |
| ≥ 65 | 832 | 1.003 (1.001-1.005) | 0.009 |  |
| Sex |  |  |  | 0.138 |
| Male | 1318 | 1.001 (0.999-1.003) | 0.255 |  |
| Female | 1355 | 1.003 (1.001-1.006) | 0.009 |  |
| **PLR** | 2673 | 1.01 (1.01-1.01) | <0.001 |  |
| Age |  |  |  | 0.127 |
| < 65 | 1841 | 1.01 (1.00-1.01) | 0.001 |  |
| ≥ 65 | 832 | 1.01 (1.01-1.02) | <0.001 |  |
| Sex |  |  |  | 0.231 |
| Male | 1318 | 1.01 (1.01-1.02) | <0.001 |  |
| Female | 1355 | 1.01 (1.00-1.01) | 0.019 |  |
| **SII** | 2673 | 1.001 (1.001-1.002) | <0.001 |  |
| Age |  |  |  | 0.057 |
| < 65 | 1841 | 1.001 (1.000-1.002) | 0.049 |  |
| ≥ 65 | 832 | 1.003 (1.001-1.004) | <0.001 |  |
| Sex |  |  |  | 0.573 |
| Male | 1318 | 1.001 (1.000-1.002) | 0.010 |  |
| Female | 1355 | 1.002 (1.001-1.003) | 0.005 |  |
| **SIRI** | 2673 | 1.361 (0.983-1.885) | 0.064 |  |
| Age |  |  |  | 0.307 |
| < 65 | 1841 | 1.082 (0.670-1.748) | 0.748 |  |
| ≥ 65 | 832 | 1.525 (0.986-2.360) | 0.058 |  |
| Sex |  |  |  | 0.085 |
| Male | 1318 | 1.002 (0.641-1.566) | 0.993 |  |
| Female | 1355 | 2.024 (1.128-3.631) | 0.018 |  |
